# Evaluation of the Relationship Between Serum Uric Acid and Cardiovascular Disease: A Cross‐Sectional Study in Bangladesh

**DOI:** 10.1002/edm2.70055

**Published:** 2025-05-01

**Authors:** Nurshad Ali, Nayan Chandra Mohanto, Ali Newaj, Jannat Begum, Farjana Islam

**Affiliations:** ^1^ Department of Biochemistry and Molecular Biology Shahjalal University of Science and Technology Sylhet Bangladesh

**Keywords:** adults, association, Bangladesh, cardiovascular disease, serum uric acid

## Abstract

**Objectives:**

Serum uric acid (SUA) has been suggested to be associated with obesity, dyslipidaemia, diabetes, and hypertension. However, whether uric acid is independently associated with the risk of myocardial infarction (MI), a major type of cardiovascular disease (CVD), remains debatable, especially across different populations. This study aims to examine the relationship between SUA levels and MI in an adult population group in Bangladesh.

**Methods:**

The study included 392 participants: 188 with a history of MI in the CVD group and 204 healthy individuals without CVD in the control group. Anthropometric, blood pressure, SUA, and other biochemical parameters were measured. A multivariate regression model was used to assess the relationship between elevated SUA levels and the risk of CVD.

**Results:**

The mean level of SUA was significantly higher in the CVD group (7.6 ± 4.5 mg/dL) compared to the non‐CVD group (5.3 ± 1.8 mg/dL) (*p* < 0.001). The prevalence of hyperuricemia was also observed to be higher in the CVD group (46.3%) compared to the non‐CVD group (18.2%) (*p* < 0.001). A significant difference was observed in the levels of blood glucose and lipid profile between the CVD and non‐CVD groups (*p* < 0.001 for all cases). No significant differences were observed in the mean level of SUA or the prevalence of hyperuricemia between the gender groups. When SUA was divided into four quartiles, a significant difference was observed for systolic blood pressure across the quartile groups. After adjusting for potential confounders in the regression models, SUA was found to have a significant association with CVD.

**Conclusions:**

Elevated levels of SUA were associated with increased odds of CVD among the study participants. Managing SUA levels and implementing intervention strategies could be effective in preventing and controlling cardiovascular events.

## Graphical abstract

After categorising the participants into different age groups, a significant difference in serum uric acid level was observed. The level of SUA and the prevalence of hyperuricemia were higher in the CVD group compared to the non‐CVD group.

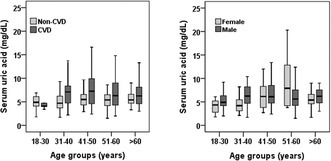

## 1 Introduction

Uric acid is produced as a result of purine metabolism, primarily in the liver, with a small amount also generated in the small intestine [1, 2]. Normally, around two‐thirds of uric acid is eliminated through the kidneys and the remaining one‐third is eliminated through the intestines [1, 2]. High levels of uric acid in the blood, known as hyperuricemia, can be caused by overproduction of uric acid or reduced renal excretion, or both [3]. Humans have a serum uric acid (SUA) concentration several times higher than other mammals due to the absence of urate oxidase, an enzyme that converts uric acid to allantoin [4]. Hyperuricemia is becoming increasingly common due to the rise in high‐protein and high‐purine foods, or high fructose beverages, in our diets, as well as sedentary lifestyles that contribute to obesity and type 2 diabetes [2, 5].

Cardiovascular disease (CVD) is the leading cause of death all over the world [6, 7]. Hypertension, diabetes, obesity, and smoking are well‐known risk factors for CVDs [8, 9]. In Bangladesh, CVDs are responsible for 30% of all deaths, while non‐communicable diseases account for 67% of all deaths [10]. Although traditional prediction models based on risk factors are useful in predicting most CVD events, they do not account for all cardiovascular risk factors [11, 12]. As a result, new biomarkers and risk factors are still being evaluated to improve CVD risk stratification [11, 12, 13].

Research indicates that uric acid might be an independent risk factor for CVDs when accounting for multiple risk factors [14, 15, 16]. However, there is no clear evidence that it is a causal factor [17]. Additionally, there is limited data on whether SUA can help improve the classification of CVD risk, particularly in individuals with intermediate risk. It has been suggested that high levels of SUA may be a compensatory response to counteract oxidative damage that is associated with atherosclerosis [18]. However, it has yet to be tested whether reducing SUA levels could prevent CVD [19]. Numerous studies have suggested that high levels of SUA pose a significant risk for CVDs [14, 15, 20]. Epidemiological studies have also indicated a potential link of hyperuricemia with hypertension, obesity, diabetes, metabolic syndrome, coronary artery disease, and cerebrovascular diseases [21, 22, 23, 24, 25, 26]. Some clinical trials suggest that lowering SUA levels may benefit the cardiovascular system [27, 28]. There are varying opinions on whether high levels of SUA are a risk factor for CVDs. Some experts in the Framingham Heart Study group believe that clinicians should only consider traditional risk factors when evaluating patients, while others hold the opposite view [29]. Furthermore, professional bodies have not yet recognised SUA as a cardiovascular risk factor [30, 31]. The increased prevalence of CVDs is the leading cause of death in Bangladesh, but the underlying causes remain unknown [32]. So far, no studies have yet investigated the potential relationship between elevated SUA levels and CVD in the Bangladeshi population. In the present study, we aimed to assess the relationship between SUA levels and CVD in an adult population group in Bangladesh. A better understanding of the clinical significance of this relationship may prompt earlier monitoring of SUA levels and may identify populations at high risk of CVD.

## 2 Methods

### 2.1 Study Area and Participants

Between February 2019 and January 2020, this cross‐sectional study was conducted to collect blood samples and data. The study involved 188 participants with MI in the CVD group recruited from Osmani Medical College Hospital, Sylhet. Additionally, 204 participants without CVD in the control group were enrolled from the general population of the Sylhet region in Bangladesh. The participants with MI were identified through the International Classification of Diseases, 10th revision (ICD‐10) codes (I21‐I22) and confirmed by baseline ECG or medical records in the baseline interview. The inclusion criteria included both sexes, participants who were older than 18 years and suffering from myocardial infarction. Pregnant women, nursing mothers, subjects previously diagnosed with hepatic diseases, renal diseases, and any infectious diseases were excluded from the study. Each subject gave their written informed consent before participating in the study. The study protocol (Reference no 02/BMB/2019) was approved by the Ethics Committee at the Department of Biochemistry and Molecular Biology, School of Life Science, Shahjalal University of Science and Technology. The study was conducted in accordance with relevant guidelines and regulations.

### 2.2 Anthropometric and Blood Pressure Data

Anthropometric measurements such as weight, height, and blood pressure were taken using standardised protocols as described elsewhere [33, 34, 35, 36, 37, 38]. The participant's weight was measured using a digital scale (Beurer 700, Germany) while wearing light clothing without shoes. Standing height was measured using a tape meter without shoes. Body mass index (BMI) was calculated as weight divided by height in meters squared (kg/m^2^). Systolic and diastolic blood pressures (SBP and DBP, respectively) were measured twice after the participants rested in a seated position for 10 min using a digital sphygmomanometer (Omron M10, Omron Corporation, Tokyo, Japan) and the results were averaged during data analysis. Additionally, the participants' physical activity and smoking status were included in the questionnaire.

### 2.3 Blood Collection and Laboratory Analysis

About 5 mL of blood samples were collected from the participants with the help of expert personnel. The blood samples were transported quickly to the clinical biochemistry laboratory. After centrifugation, serum samples were separated and stored at a temperature of −20°C until targeted markers analysis. The diagnostic kits were purchased from HUMAN Gesellschaft für Biochemica und Diagnostica mbH, Germany. Biochemical markers like serum uric acid (SUA), fasting blood glucose (FBG), total cholesterol (TC), triglycerides (TG), high‐density lipoprotein cholesterol (HDL‐C), and low‐density lipoprotein cholesterol (LDL‐C) were measured using a colorimetric method and a semi‐automatic biochemistry analyser (Humalyzer 3000, Medicon Services, Germany). The measurements were carried out according to the standard protocols provided within the kit, and we ensured the precision of the measurements by maintaining method standard calibration on a regular basis.

### 2.4 Diagnostic Criteria

The diagnosis of CVD (specifically myocardial infarction or MI) is determined according to the International Classification of Diseases, 10th revision (ICD‐10) codes (I21‐I22), and confirmed by baseline ECG or medical records in the baseline interview. Hypertension is defined as a SBP of ≥ 140 mmHg and/or DBP of 90 mmHg, or self‐reported use of antihypertensive medications [30]. Participants with diabetes are identified by checking prescriptions provided by physicians and/or self‐reported use of anti‐diabetic medications. Diabetes is defined according to the American Diabetes Association 2020 as a fasting blood plasma glucose level of 126 mg/dL (7 mmol/L) or higher, non‐fasting plasma glucose of 200 mg/dL (11.1 mmol/L) or higher, recent self‐reported use of insulin or hypoglycaemic drugs [39]. Hyperuricemia is defined as SUA level exceeding 7.0 mg/dL in males and 6.0 mg/dL in females [21, 22]. Physical activity is classified into three groups: inadequate (comfortable office work and housework), medium (walking, swimming), and adequate (carrying, lifting, jogging, and/or sports). Smoking status is divided into never smokers or current smokers.

### 2.5 Statistical Data Analysis

The data was presented as mean ± SD and quartile ranges. An independent sample t‐test was used to compare the baseline characteristics of the volunteers in the gender group. One‐way ANOVA was used for the SUA quartiles. A Chi‐square test was applied to differentiate the proportions of the categorical variables. To assess the association between SUA levels and CVD, multivariate logistic regression models were used. CVD was categorised as “yes” (presence) or “no” (absence). In the regression analysis, CVD (yes) was considered the dependent variable, while SUA served as the independent variable. Three models were used in the regression analysis. Model 1 was adjusted for age (and sex for overall). Model 2 was adjusted for variables in model 1 + BMI, SBP, DBP, and glucose. Model 3 was adjusted for variables in model 2 + lipid markers (TG, TC, HDL, and LDL), physical activity, and smoking status. All statistical results were analysed using IBM SPSS version 23, and P‐values below 0.05 are considered statistically significant.

## 3 Results

### 3.1 Characteristics of the Study Subjects

**FIGURE 1:**
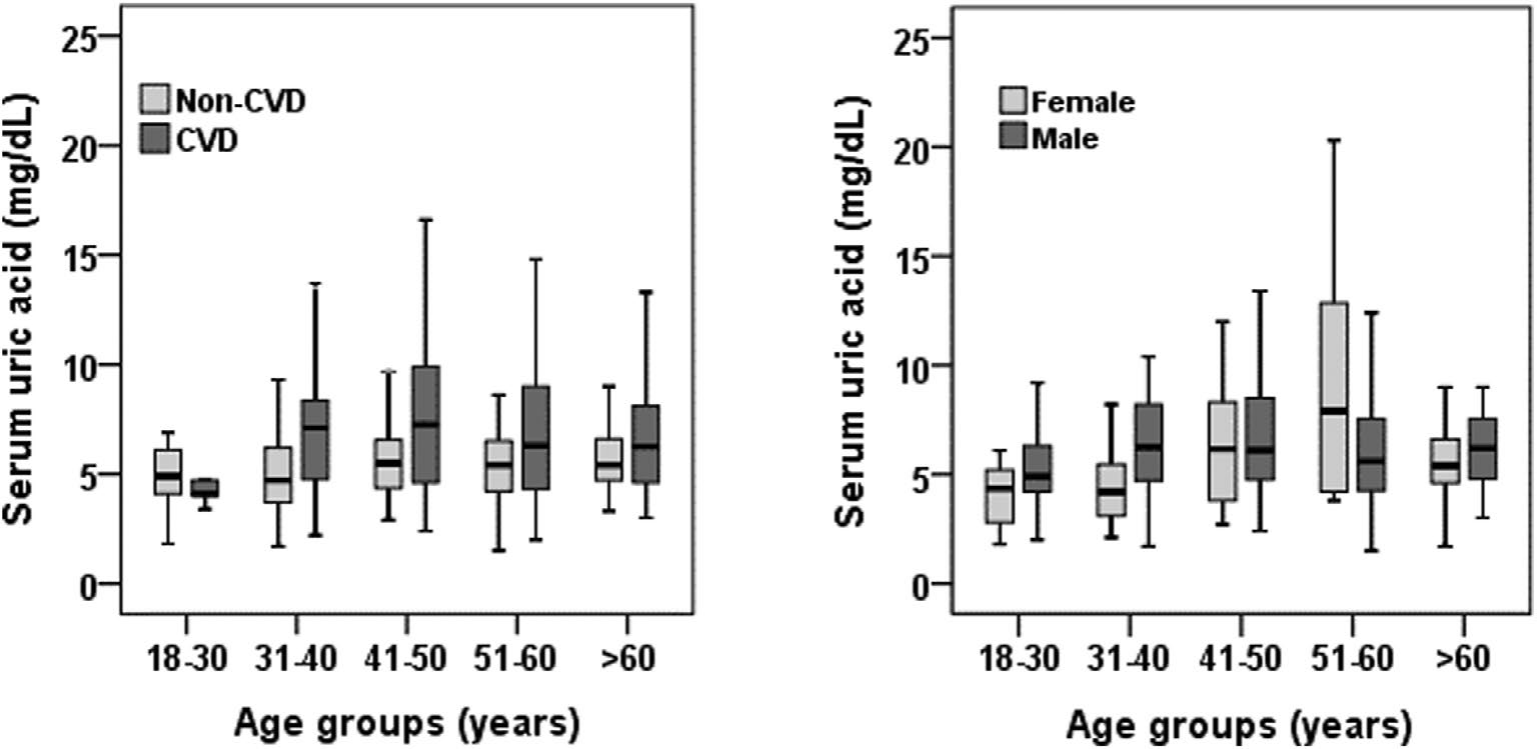
Levels of SUA in different age groups according to CVD prevalence and sex groups. *p* < 0.01 when the levels of SUA are compared within the age groups. *p*‐values are obtained from one‐way ANOVA.

Table 1 presents the characteristics of the study participants. Out of the total participants, 287 were male and 105 were female. The mean age of all participants was 48.5 ± 13.8 years, with a significant difference between the CVD and control groups (52.3 ± 11.2 years and 43.8 ± 15.0 years, respectively) (*p* < 0.001). The control group had a higher BMI level (24.2 ± 3.8 kg/m^2^) than the CVD group (21.4 ± 3.1 kg/m^2^) (*p* < 0.001). The mean level of SUA was significantly higher in the CVD group (7.6 ± 4.5 mg/dL) than in the control group (5.3 ± 1.83 mg/dL) (*p* < 0.001). The prevalence of hyperuricemia was higher in the CVD (46.3%) group than in the non‐CVD (18.2%) group (*p* < 0.001). There was also a significant difference in blood pressure, serum levels of glucose and lipid profile (TG, TC, HDL‐C, and LDL‐C) between the CVD and control groups (*p* < 0.001 for all cases). After dividing the participants into different age groups according to gender and CVD prevalence, significant differences were found in the levels of SUA within the groups (*p* < 0.01) (Figure 1). Furthermore, significant differences were observed in smoking status and physical activity between the CVD and control groups (*p* < 0.001).

**TABLE 1:** Baseline characteristics of the participants in control case groups.

Variable	Overall	Non‐CVD	CVD	*p*
Number (*N*)	392	204	188	—
Gender (f/m)	105/287	65/139	40/148	—
Age (years)	48.5 ± 13.8	43.9 ± 15.0	52.3 ± 11.2	0.000
BMI (kg/m^2^)	22.6 ± 3.7	24.2 ± 3.8	21.4 ± 3.1	0.000
SBP (mm Hg)	123.4 ± 22.6	130.9 ± 18.4	117.3 ± 23.9	0.000
DBP (mm Hg)	78.1 ± 12.6	80.9 ± 9.8	75.7 ± 14.1	0.000
Glucose (mmol/L)	6.7 ± 3.6	7.7 ± 4.3	6.0 ± 2.5	0.000
Creatinine (mg/dL)	0.94 ± 0.61	0.90 ± 0.36	0.97 ± 0.76	0.359
SUA (mg/dL)	6.6 ± 3.7	5.3 ± 1.8	7.6 ± 4.5	0.000
TG (mg/dL)	165.7 ± 105.8	197.3 ± 124.9	139.6 ± 78.0	0.000
TC (mg/dL)	180.3 ± 78.6	224.1 ± 90.0	144.3 ± 41.5	0.000
HDL‐C (mg/dL)	26.9 ± 15.5	34.9 ± 15.8	20.2 ± 11.8	0.000
LDL‐C (mg/dL)	120.0 ± 68.8	149.8 ± 82.3	95.6 ± 41.9	0.000
Hyperuricaemia (%)	33.6	18.2	46.3	0.000
Diabetes (%)	33.9	34.4	31.8	0.361
Hypertension (%)	32.6	35.5	30.5	0.066
Smoking (%)				
Yes	50.0	28.6	67.6	0.000
No	50.0	71.4	32.4	
Physical activity (%)				
Low	41.9	19.6	60.1	0.000
Medium	51.3	69.9	36.2	
High	6.7	10.5	3.7	

*Note:* Data are presented as mean ± SD. *p*‐values are obtained from independent sample *t*‐test in comparison between control (Non‐CVD) and case (CVD) groups.

Abbreviations: BMI, body mass index; DBP, diastolic blood pressure; HDL‐C, high‐density lipoprotein cholesterol; LDL‐C, low‐density lipoprotein cholesterol; SBP, systolic blood pressure; SUA, serum uric acid; TC, total cholesterol; TG, triglyceride.

### 3.2 Subject's Characteristics by Gender and SUA Quartiles

Table 2 presents the baseline characteristics based on sex. In the CVD group, both males and females had a higher mean age than the control group (*p* < 0.001). Females had a higher BMI and blood pressure than males in both groups (*p* < 0.001). The mean SUA was higher among females than male participants. For both genders, a significant difference was observed in the blood glucose and lipid profile between the CVD and control groups (at least *p* < 0.01), and females typically had a higher mean level of the biochemical parameters. Though the difference was not statistically significant, the prevalence of hypertension and diabetes was higher among females than their counterparts. Among the study participants, only males smoked, with 40.4% in the control group and 81.1% in the CVD group. The control group had a higher level of physical activity than the CVD group for both genders (*p* < 0.001), and males were more active than females. When SUA levels were divided into four quartiles: Q1 (< 4.3 mg/dL), Q2 (4.4–5.6 mg/dL), Q3 (5.7–7.7 mg/dL), and Q4 (> 7.7 mg/dL), a significant increasing trend was observed for systolic blood pressure (SBP) across the SUA quartiles (*p* < 0.05) (Table 3). Additionally, a significant trend was found for serum creatinine, HDL‐C, and physical activity (with at least *p* < 0.05 for all cases).

**TABLE 2:** Baseline characteristics of the participants according to sex.

Variable	Female			Male		
	Non‐CVD	CVD	*p*	Non‐CVD	CVD	*p*
Number (*N*)	65	40	—	139	148	—
Age (years)	41.3 ± 16.3	54.7 ± 11.9	0.000	45.0 ± 14.6	51.7 ± 11.0	0.000
BMI (kg/m^2^)	24.5 ± 4.0	20.2 ± 3.3	0.000	24.0 ± 3.8	21.7 ± 3.0	0.000
SBP (mm Hg)	130.1 ± 22.0	115.8 ± 27.4	0.009	131.2 ± 16.8	117.7 ± 23.0	0.000
DBP (mm Hg)	82.0 ± 9.6	74.4 ± 17.3	0.013	80.6 ± 9.8	76.1 ± 13.2	0.003
Glucose (mmol/L)	7.4 ± 3.6	5.8 ± 2.4	0.019	7.8 ± 4.6	6.0 ± 2.6	0.000
Creatinine (mg/dL)	0.68 ± 0.18	0.87 ± 0.53	0.025	1.00 ± 0.38	0.99 ± 0.81	0.953
SUA (mg/dL)	4.3 ± 1.4	8.2 ± 4.9	0.000	5.7 ± 1.8	7.5 ± 4.4	0.000
TG (mg/dL)	135.3 ± 77.8	118.2 ± 58.0	0.257	222.9 ± 131.9	145.5 ± 81.8	0.000
TC (mg/dL)	235.5 ± 102.8	158.8 ± 48.0	0.000	219.4 ± 84.2	140.3 ± 38.8	0.000
HDL‐C (mg/dL)	34.6 ± 8.9	21.6 ± 8.4	0.000	35.0 ± 17.9	19.9 ± 12.5	0.000
LDL‐C (mg/dL)	173.8 ± 102.0	113.5 ± 50.6	0.001	139.9 ± 70.8	90.7 ± 38.0	0.000
Hyperuricaemia (%)	8.9	62.5	0.000	22.0	49.9	0.000
Diabetes (%)	36.6	31.0	0.586	34.9	30.7	0.472
Hypertension (%)	36.3	29.5	0.268	34.1	30.8	0.141
Smoking (%)						
Yes	0.0	17.5	0.003	40.4	81.1	0.000
No	100.0	82.5		59.6	18.9	
Physical activity (%)						
Low	24.4	80.0	0.000	17.6	54.7	0.000
Medium	71.1	20.0		69.4	40.5	
High	4.4	0.0		13.0	4.7	

*Note:* Data are presented as mean ± SD. *p*‐values are obtained from independent sample *t*‐test in comparison between control (Non‐CVD) and case (CVD) groups.

Abbreviations: BMI, body mass index; DBP, diastolic blood pressure; HDL‐C, high‐density lipoprotein cholesterol; LDL‐C, low‐density lipoprotein cholesterol; SBP, systolic blood pressure; SUA, serum uric acid; TC, total cholesterol; TG, triglyceride.

**TABLE 3:** Baseline characteristics of the study participants according to SUA quartiles.

	Q1 (≤ 4.3 mg/dL)	Q2 (4.4–5.6 mg/dL)	Q3 (5.7–7.7 mg/dL)	Q4 (> 7.7 mg/dL)	*p*‐values for trend
*N*	103	98	93	98	—
Sex (f/m)	39/64	24/74	18/75	24/74	—
Age (years)	46.8 ± 14.0	48.2 ± 15.1	48.5 ± 14.7	50.7 ± 10.8	0.288
BMI (kg/m^2^)	22.7 ± 4.1	22.8 ± 3.4	23.0 ± 3.6	21.8 ± 3.7	0.169
SBP (mm Hg)	122.2 ± 20.9	124.5 ± 21.6	128.9 ± 23.3	118.6 ± 23.9	0.029
DBP (mm Hg)	78.4 ± 11.9	78.4 ± 12.5	80.3 ± 13.5	75.4 ± 12.2	0.090
Glucose (mmol/L)	7.2 ± 4.2	6.6 ± 3.7	6.5 ± 3.3	6.5 ± 2.9	0.422
Creatinine (mg/dL)	0.83 ± 0.39	0.85 ± 0.30	1.12 ± 0.98	0.97 ± 0.61	0.005
SUA (mg/dL)	3.43 ± 0.75	5.0 ± 0.37	6.6 ± 0.60	11.5 ± 4.17	0.000
TG (mg/dL)	154.5 ± 98.0	187.2 ± 119.9	162.3 ± 95.1	159.6 ± 107.3	0.181
TC (mg/dL)	185.6 ± 75.4	183.2 ± 67.5	191.2 ± 92.8	161.7 ± 75.7	0.077
HDL‐C (mg/dL)	25.4 ± 12.3	31.4 ± 19.3	27.9 ± 13.7	22.9 ± 15.0	0.003
LDL‐C (mg/dL)	129.2 ± 66.3	113.0 ± 55.1	130.7 ± 85.6	106.9 ± 64.0	0.055
Hyperuricaemia (%)	0.0	0.0	37.5	100.0	0.000
Diabetes (%)	34.7	29.2	28.5	38.6	0.446
Hypertension (%)	32.0	30.5	39.2	28.8	0.363
Smoking (%)					
Yes	42.4	50.6	50.0	57.6	0.248
NO	57.6	49.4	50.0	42.4	
Physical activity (%)					
Low	37.4	37.6	35.0	57.6	0.011
Medium	50.5	56.5	60.0	38.8	
High	12.1	5.9	5.0	3.5	

*Note:* Values are presented as mean ± SD. *p*‐values are obtained from one‐way ANOVA.

Abbreviations: BMI, body mass index; DBP, diastolic blood pressure; HDL‐C, high‐density lipoprotein cholesterol; LDL‐C, low‐density lipoprotein cholesterol; SBP, systolic blood pressure; SUA, serum uric acid; TC, total cholesterol; TG, triglyceride.

### 3.3 Association Between SUA and CVD

The relationship between SUA levels and CVD is presented in Table 4. In order to evaluate the relationship, three models were applied in logistic regression analysis. After adjusting for age, sex, and other variables in the regression models, the odds ratios (ORs) and 95% CI were 1.268 (1.153–1.395), 1.330 (1.182–1.495), and 1.360 (1.152–1.606) respectively in Q1 to Q3. In all models, elevated SUA levels showed a significant and independent association with the prevalence of CVD (*p* < 0.001). When we checked this association on a gender basis, a significant association was observed for both genders; however, an increased OR was observed in females in all three models.

**TABLE 4:** Multivariate regression analysis to evaluate the association between SUA and CVD.

	*B*	SE	Wald	df	OR (95% Cl)	*p*
Overall						
Model 1	0.243	0.049	24.781	1	1.275 (1.159–1.403)	0.000
Model 2	0.299	0.061	24.292	1	1.348 (1.197–1.518)	0.000
Model 3	0.337	0.089	14.380	1	1.401 (1.177–1.668)	0.004
Female						
Model 1	0.532	0.153	12.040	1	1.702 (1.260–2.298)	0.001
Model 2	0.895	0.264	11.488	1	2.448 (1.459–4.108)	0.001
Model 3	0.873	0.418	4.352	1	2.394 (1.054–5.436)	0.037
Male						
Model 1	0.180	0.051	12.686	1	1.198 (1.085–1.323)	0.000
Model 2	0.217	0.062	12.247	1	1.242 (1.100–1.403)	0.000
Model 3	0.297	0.097	9.348	1	1.346 (1.113–1.629)	0.002

*Note:* Association between SUA levels and CVD were determined by multivariate logistic regression. The dependent variable was CVD (yes) and the independent variable was SUA (mg/dL). The reference category is control (No‐CVD). Model 1: adjusted for age (and sex for overall). Model 2: model 1 + BMI, SBP, DBP, glucose and creatinine. Model 3: model 2 + lipid markers (TG, TC, HDL‐C, and LDL‐C), physical activity and smoking status.

Abbreviations: CI, confidence interval; OR, odds ratio; SE, standard error.

## 4 Discussion

In this study we evaluated the relationship of elevated SUA with CVD in a population group from Bangladesh. Our findings showed that hyperuricemia was more prevalent in participants with MI in the CVD group compared to those who did not have CVD in the control group. In regression analysis, SUA showed a positive and independent association with CVD. The prevalence of hyperuricemia and CVD is increasing every year [40, 41]. Several studies have investigated the relationship between SUA and CVD, but the findings are still controversial. Most studies have indicated that high levels of SUA are associated with various CVDs, including coronary heart disease (CHD), stroke, congestive heart failure, and an increased risk of mortality from CVD (reviewed in [42, 43]).

Studies on the link between uric acid levels and in‐hospital mortality after an acute myocardial infarction (AMI) have produced conflicting results [44, 45]. While some studies have found a clear association between elevated uric acid levels and poor clinical outcomes (53, 54 from Demiry et al.), others have failed to find a link [46]. The Rotterdam Study, followed up for 8.4 years, reported an association between higher SUA levels and an increased risk of CVDs (MI and stroke), even after adjusting for age and sex [47]. Another study showed that SUA is an independent risk factor for fatal MI, particularly among women, after accounting for potential confounding variables [48]. The study also revealed that it's possible to identify a prognostic cut‐off value associated with fatal MI [48]. According to Kojima et al., patients with hyperuricemia who suffer from AMI are more likely to experience left ventricular systolic and diastolic dysfunction, as well as major adverse cardiovascular events such as heart failure and death [49]. This may be due to the fact that uric acid can increase oxidative stress and inflammation, leading to cardiomyocyte apoptosis and further myocardial remodelling. Reduced oxygen supply during an AMI triggers xanthine oxidase and increases SUA and oxidative stress. Elevated SUA can cause harm by initiating oxidative stress and inflammation, reducing NO production, and causing endothelial dysfunction, ultimately leading to poorer outcomes [42]. However, uric acid's antioxidant properties may provide some benefits during an AMI, but conflicting findings have been reported [42].

Further studies have investigated the role of SUA in CVD risks and mortality. For example, in a part of the Framingham study, hyperuricemia was found to be associated with an increased risk of coronary heart disease in men aged 30–59 years [40]. A study on adults aged 25–74 years noted that elevated levels of uric acid were positively associated with CHD‐related mortality [14]. In a study of Asians aged 35 years and above, hyperuricemia was found to be associated with an increased risk of all‐cause mortality, total CVD, and stroke risk by 16%, 39%, and 35%, respectively [50]. In our study, after controlling for potential confounding factors, SUA was significantly associated with MI in both genders. However, some studies have failed to show a significant link between high levels of hyperuricemia and cardiovascular events or mortality. For instance, the Framingham Cardiovascular study found no relationship between elevated uric acid levels and CVD or all‐cause mortality, after adjusting for confounding factors such as age and traditional cardiovascular risk factors, in both genders [29]. Similarly, the Third National Health and Nutrition Examination Survey (NHANES III) study found no association between SUA levels and CVD or CHD‐related mortality [51] Therefore, the connection between uric acid levels and CVD risks is still not well explained.

In our study, a significant association was observed between SUA and CVD in both genders after adjustment of confounding variables. However, increased risk (ORs) was observed in females than in the male subjects. Individuals with hyperuricemia have a significantly higher risk of CVD mortality compared to those with normal levels of uric acid [52]. Studies have shown that the association between hyperuricemia and CVD risk is especially high in women [52, 53]. In the NHANES I study, the results indicated that an increase in SUA is associated with CHD‐related mortality in both genders [14]. However, females had a higher risk of 3.00 (95% CI: 1.45–6.28) compared to males with a risk of 1.77 (95% CI: 1.08–3.980) for 4th vs. 1st UA quartiles [14]. Additionally, a meta‐analysis revealed that every 1 mg/dL increase in SUA raised the risk of CHD mortality in women (RR = 2.44), but not in men [53]. This difference might be due to the cardiovascular damage caused by uric acid. Experimental and clinical studies have shown that elevated levels of uric acid can have harmful effects on cardiovascular health. These effects include increased oxidative stress, decreased availability of nitric oxide, and endothelial dysfunction [54, 55, 56]. Uric acid can also promote local and systemic inflammation, vasoconstriction, proliferation of vascular smooth muscle cells, insulin resistance, and metabolic dysregulation [57, 58].

Vascular risk factors vary between women and men in prevalence, and evidence supports the clinical significance of sex differences in CVDs like stroke [59]. In females, elevated uric acid levels may have a greater impact on the cardiovascular system as the protective effect of decreasing oestrogen secretion decreases with age [60]. Moreover, women have longer lifespans and are more susceptible to depression, anxiety, and stress, which might lead to differences [61]. Conversely, in a study, the association between uric acid and CVD risk was observed only in males [62]. It was found that males with high levels of uric acid in the highest quartile had an increased risk of CVD by 2.55 (1.41–4.62) after adjusting for the confounders. This could be because uric acid levels were calculated as quartiles and only men's quartiles were close to the hyperuricemia criteria. Additionally, the study had a limited number of participants with moderate to high CVD risk.

Uric acid plays a dual role as both a pro‐oxidant and an antioxidant. Studies have shown that hyperuricemia can promote the development of CVD by causing endoplasmic reticulum stress, oxidative stress, insulin resistance, and endothelial dysfunction (reviewed in [56]). Although uric acid can act as a scavenger of free radicals and singlet oxygen, high levels of uric acid can lead to endothelial dysfunction and increase platelet adhesion, potentially leading to a cascade of coagulation that stimulates thrombus formation and arterial occlusion, ultimately resulting in intracranial atherosclerosis [63, 64]. Several studies have also linked uric acid to hypertension and metabolic syndrome, which can increase the risk of CVDs [22, 23, 56].

This study has a few limitations that should be taken into account when interpreting its results. Firstly, we did not have access to all the medical records of the CVD patients concerning potential confounders. For example, we lacked information on the use of medications that may lower uric acid levels, the duration of the disease, dietary habits rich in purines, and the patient's renal function. Furthermore, other potential factors such as ethnicity, genetics, and environmental factors might influence SUA metabolism and CVD risk. Therefore, the outcomes should be interpreted with caution. Secondly, the causal relationship between hyperuricemia and CVD could not be established due to the cross‐sectional nature of the study. To gain a clearer understanding of the relationship between serum uric acid levels and CVD, further longitudinal studies are necessary. However, this study effectively analysed data that included key confounding variables, which resulted in strong statistical power. Thirdly, the sample size of the study was relatively small; therefore, our findings may not be representative of the entire population of Bangladesh. Despite these limitations, our findings would be valuable for future studies.

## 5 Conclusion

The prevalence of hyperuricemia was significantly higher in participants with CVD compared to those without CVD. Elevated SUA levels were significantly associated with CVD in the study population. Our findings broaden previous research exploring the potential link between hyperuricemia and CVD while accounting for various confounding factors. Early monitoring and management of SUA levels could be beneficial for the prevention and control of cardiovascular events.

## Author Contributions

N.A. contributed to the conception and design of the study, data interpretation, drafting, and revision of the manuscript. N.C.M. and A.N. collected the samples and conducted the experiments. F.I. helped in the result analysis and revision of the manuscript draft. All authors read the manuscript and approved the final version.

## Ethics Statement

The study protocol (Ref no 02/BMB/2019) was approved by the Ethics Committee at the Department of Biochemistry and Molecular Biology, School of Life Science, Shahjalal University of Science and Technology.

## Conflicts of Interest

The authors declare no conflicts of interest.

## Data Availability

The data that support this study's findings are available from the corresponding author upon reasonable request.
